# Cannabis Use Among Veterans: It Should be Easier to Get Some

**DOI:** 10.1093/geroni/igab046.1967

**Published:** 2021-12-17

**Authors:** Julie Bobitt

**Affiliations:** Center for Dissemination and Implementation Science, University of Illinois at Chicago, Illinois, United States

## Abstract

and PTSD. From December 2020 – February 2021 we conducted 32 semi-structured interviews with Veterans who responded to our initial and follow-up surveys and agreed to discuss their cannabis use. We coded and themed the interviews using inductive thematic analysis. We found that many Veterans are using cannabis in place of other medications such as opioids and benzodiazepines and often do so to avoid the negative side effects. However, barriers such as Veterans Administration policies and cost of medical cannabis affect Veterans ability to obtain medical cannabis. Our results inform clinicians and policy makers on the use of cannabis as an alternative to prescription medications for treating chronic pain and other conditions in older Veterans.

